# Analytic posterior distribution and bayes factor for pearson partial correlations

**DOI:** 10.1007/s11749-026-01006-x

**Published:** 2026-06-02

**Authors:** Šimon Kucharský, Eric-Jan Wagenmakers, Don van den Bergh, Alexander Ly

**Affiliations:** 1 Department of Psychology, University of Amsterdam, Amsterdam, The Netherlands; 2 Department of Statistics, TU Dortmund, Dortmund, Germany; 3 Centrum Wiskunde & Informatica, Amsterdam, The Netherlands

**Keywords:** Bayes factor, Posterior distribution, Partial correlation, 62F15, 62H20, 62H10

## Abstract

This article outlines a novel Bayesian approach to the testing and estimation of Pearson partial correlations. By generalizing a Bayesian inference procedure for Pearson’s correlation coefficient, we obtain analytic expressions for the Bayes factor and for the (marginal) posterior distribution of a partial correlation coefficient. Full Bayesian inference can be achieved using only the sample size, the number of controlling variables and the relevant summary statistics, that is, the sample partial correlation. The present approach is illustrated with two empirical examples.

## Introduction

The Pearson partial correlation coefficient quantifies the linear relationship between two continuous variables while taking into account the effects of other (confounding) variables. Under certain distributional assumptions (e.g., multivariate normality), the partial correlation coincides with a conditional correlation and can therefore be used to assess conditional (in)dependence between a set of variables (Baba et al. 2004; Baba and Sibuya 2005; Lawrance 1976). This fact is central in Gaussian Graphical Models (Lauritzen 1996), where partial correlations are used to map out unique relationships between a number of variables (i.e., partial correlation networks; Costantini et al. 2015). For these reasons, inference for partial correlation is included both in popular introductory statistical textbooks (Agresti and Finlay 2009; Field 2017; Lomax and Hahs-Vaughn 2012; Moore et al. 2012) and statistical software packages (e.g., Kim 2015; Jasp Team 2020; Ibm Corp 2017) as a basic statistical tool.

In practical applications, researchers often wish to test whether a population partial correlation is zero. The dominant approach is to use frequentist null hypothesis significance testing, which is already well developed for the case of a partial correlation (Weatherburn 1961, pp. 242–263). However, frequentist hypothesis testing comes with several limitations (e.g., Wagenmakers 2007; Amrhein et al. 2019; Nuzzo 2014; Wasserstein and Lazar 2016), one of them being the inability to quantify evidence in favor of the null hypothesis. In other words, the frequentist test does not discriminate between ‘evidence of absence’ and ‘absence of evidence’ (e.g., Keysers et al. 2020). This is particularly problematic for partial correlations, because researchers often wish to claim evidence for the null hypothesis of conditional independence. In general, it is desirable that a method of testing can quantify evidence in favor of either conditional independence or conditional dependence (Epskamp 2017, pp. 240–241).

This frequentist limitation can be overcome within the framework of Bayesian statistics, namely with *Bayes factors* (Jeffreys 1961; Kass and Raftery 1995). Bayes factor tests for partial correlations have already been proposed by Wetzels and Wagenmakers (2012), Wang et al. (2019), Williams and Mulder (2020), and Mulder and Gelissen (2023). First, Wetzels and Wagenmakers (2012) proposed to test the coefficient indirectly by testing an increase of explained variance in a linear regression model. However, this procedure yields results that are sensitive to the direction of the effect (i.e., which of the two variables of interest is used as the predictor), which is undesirable as partial correlation is an undirected coefficient. Second, Wang et al. (2019) also use a setup from linear regression with the popular Zellner’s g-prior (Zellner 1986), but proposed to test the regression coefficient instead, which effectively leads to converting the coefficient to a *t*-statistic. One downside of this approach is that the resulting Bayes factor inherits an issue of the Zellner’s g-prior in that it is not information consistent (Liang et al. 2008). A drawback common to both approaches is that they do not test the partial correlation directly, but represent it with a proxy statistic, be it a change in $R^2$ or a coefficient in a linear regression. This makes it difficult to reason about the implied prior distribution on the partial correlation coefficient, frustrating the use of informed priors. Neither approach provides the posterior distribution for the population partial correlation coefficient, which is of course the central target for Bayesian parameter estimation.

A third Bayes factor test for partial correlations was introduced by Williams and Mulder (2020) and Williams (2021), who proposed to fit a full multivariate normal model to the data with either Wishart or Matrix-F prior distributions Mulder and Pericchi (2018) on the covariance matrix. Based on the full sample covariance matrix, it is then possible to obtain Bayes factors for individual partial correlations through the Savage-Dickey density ratio Dickey and Lientz (1970). To obtain the Bayes factor, Williams and Mulder (2020) proposed to use either analytic approximations to the posterior distributions, or apply MCMC sampling. This method is available to the practical researcher with the R package BGGM (Williams and Mulder 2019). In addition to these approaches, Mulder and Gelissen (2023) proposed a Bayes factor methodology for testing hypotheses on measures of association, including Pearson correlations and partial correlations. Their method is based on specifying a uniform prior on the parameter space under each (possibly constrained) hypothesis. Given this prior specification, the Bayes factor is obtained from the ratio of the prior and posterior probabilities that the parameter satisfies the constraint defining the hypothesis (e.g., equality, inequality, or order constraints). The approach is general and allows testing single correlations or partial correlations, as well as more complex hypotheses such as ordering restrictions, and is available in an R package BFpack (Mulder et al. 2021) and in a user-friendly statistical software JASP (Mulder et al. 2025).

Here we introduce a new Bayesian approach to test and estimate partial correlation coefficients. Our work generalizes the Bayesian development for Pearson’s correlation coefficient (Ly et al. 2016b, 2018), and inherits many of its desirable properties. For instance, the complete Bayesian inference can be conducted with only the relevant summary statistics and is computationally cheap. Our main results are twofold and address both Bayes factor testing and Bayesian parameter estimation. First, we provide an expression for the Bayes factor of a nullity of a partial correlation coefficient. We elaborate how the proposed Bayes factor fulfils certain desiderata that allow intuitive inferences, making it an attractive option for a default Bayesian testing method (Bayarri et al. 2012; Jeffreys 1961; Ly et al. 2016b). Second, we derive an analytic marginal posterior distribution for the partial correlation coefficient. This posterior distribution facilitates a Bayesian estimation effort. In general, the inference is carried out on the partial correlation coefficient itself which also encourages the use of informed prior distributions (e.g., Gronau et al. 2020). Table 1 provides an overview of the existing methods.

**Table 1:** Overview of the existing methods for partial correlation inference

Method	Inference target	Advantages	Limitations
Frequentist (e.g., Weatherburn 1961)	*p*-value and/or CIs of partial correlation coefficient	Easy to calculate, requires only summary statistics	Cannot express evidence in favor of null, among other downsides of frequentist approaches
Wetzels and Wagenmakers (2012)	BF of change in $R^2$ in linear regression	Easy to calculate	Not direction invariant, no posterior for the partial correlation
Wang et al. (2019)	BF of a regression coefficient with *g*-prior	Easy to calculate	Not information consistent, no posterior for the partial correlation
Williams and Mulder (2020); Williams (2021)	Implied BF, posterior from a full multivariate normal model with Wishart / Matrix-F priors	Inference for all partial correlations in one graph at once	Approximate, potentially computationally more demanding, requires full data or covariance matrix
Mulder and Gelissen (2023)	Bayes factors for equality, inequality, or order constraints on (partial) correlations via uniform priors	Flexible, general approach, Bayes factors and posteriors	Approximate, potentially computationally more demanding, cannot use other than uniform prior
Bayesian (this paper)	Bayes factor for nullity of partial correlation; stretched beta prior	Analytic Bayes factor and marginal posterior, computationally efficient, predictive matching and information consistency, requires only summary statistics	Evaluating Gaussian hypergeometric function

The paper continues as follows: Sect. 2 presents the proposed Bayes factor and (marginal) posterior for the partial correlation coefficient explicitly. Section 3 highlights two properties of the proposed Bayes factor. Section 4 illustrates the method with two examples, and the paper is concluded in Sect. 5.

## Bayesian inference for a partial correlation

**Fig. 1:**
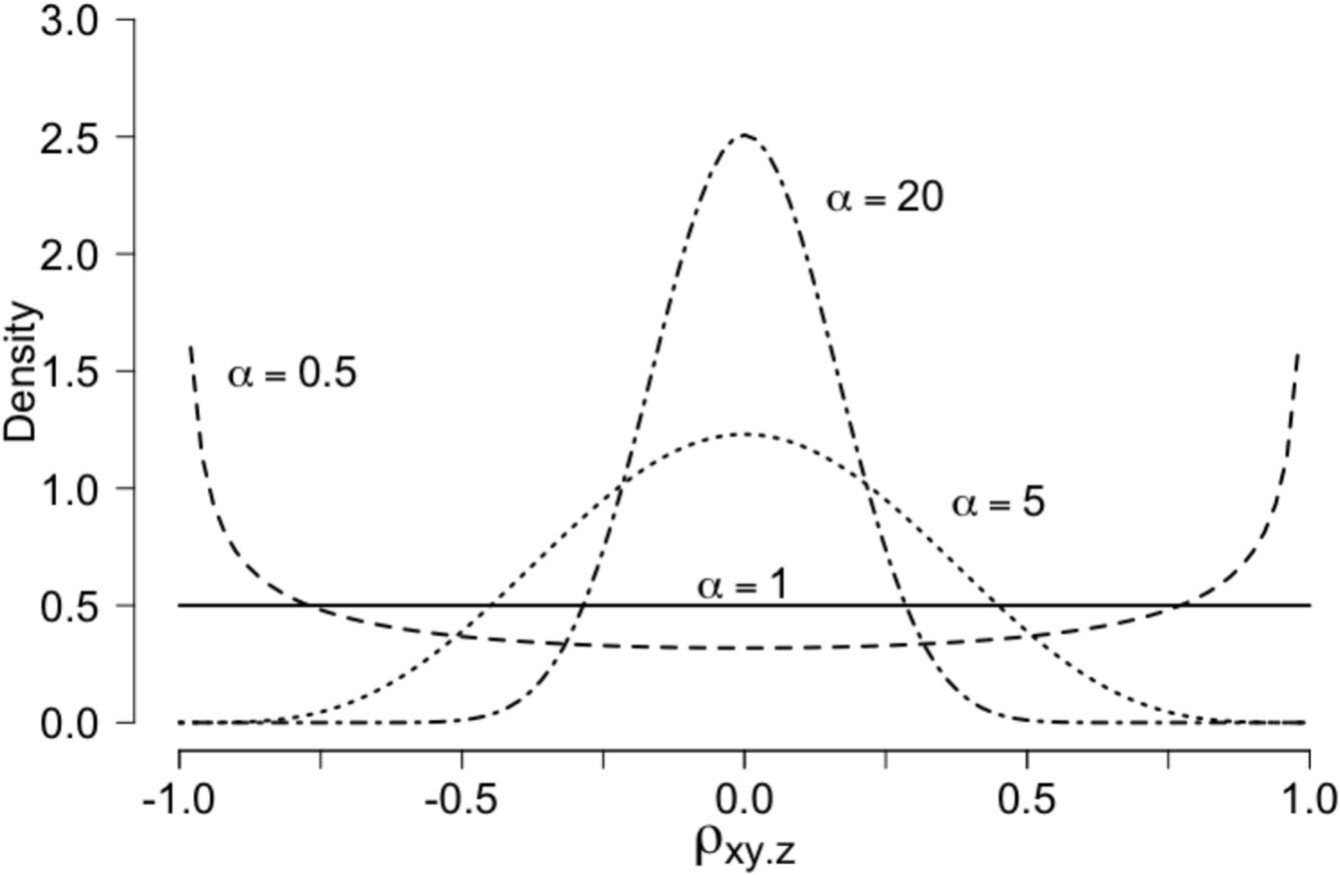
Examples of the symmetric stretched beta prior distribution for the partial correlation $\rho _{xy.z}$ defined by four different values for the hyperparameter α

Interest centers on the population partial correlation $\rho _{xy.z}$ that measures the degree of association between *X* and *Y* after the effects of *k* number of controlling variables *Z* on *X* and *Y* are removed. We focus on two somewhat different aspects of Bayesian inference: testing (i.e., “is there evidence for the presence of an effect?”) and estimation (i.e., “assuming the effect exists, how strong is it?”). For testing, the relevant question is whether or not the partial correlation equals zero – more specifically, whether and to what extent the data provide support for (or against) the hypothesis that the partial correlation coefficient equals zero. To address this question we may report the Bayes factor that compares the predictive performance of the null model $\mathcal {M}_0$ that operationalizes the null hypothesis $\rho _{xy.z} = 0$ to the predictive performance of the alternative model $\mathcal {M}_1$ that operationalizes the alternative hypothesis $\rho _{xy.z} \in (-1, 1)$. For estimation, the alternative model is assumed to hold true, wherein an unknown (population) partial correlation is free to vary between -1 and 1. The goal is then to infer this unknown parameter based on the available data. To address this question we report the posterior distribution of the partial correlation.

In Appendix A, we provide the detailed derivations of the Bayes factor and posterior distribution presented in this section. It is assumed that the variables *X*, *Y* and the *k* number of controlling variables *Z* are jointly multivariate-normally distributed. The resulting p=k+2 multivariate normal distribution has p(p+3)/2 number of parameters, but the focus of inference is only on one of them, namely, $\rho _{xy.z}$. For instance, for k=0,1,2,3,4 controlling variables there are 5, 9, 14, 20 and 27 parameters, respectively, thus, 4, 8, 13, 19 and 26 so-called nuisance parameters. Analogously, the data from a *p* multivariate normal distribution can be (sufficiently) summarized by p(p+3)/2 values. Our derivations show that the use of specific priors result in an analytic Bayes factor and marginal posterior for $\rho _{xy.z}$ that solely depend on the sample size, the number of controlling variables, and the corresponding sample partial correlation $r_{xy.z}$ from the total of p(p+3)/2 sufficient statistics. This is achieved in Appendix A by (1) isolating that part of the likelihood that only involves $\rho _{xy,z}$ and its sampled counterpart $r_{xy.z}$, and by (2) choosing appropriate priors on the nuisance parameters.

Isolating the parameter $\rho _{xy.z}$ is achieved by re-parametrizing the multivariate normal likelihood by Schur decomposition of the variance-covariance matrix Σ into three parameters $\Sigma _{11.2}$, the covariance matrix of the residuals of variables *X* and *Y* after regressing them on *Z*, $\Sigma _{22}$, the covariance matrix of the conditioning variables *Z*, and $\textrm{B}$, the matrix of regression coefficients. Further, $\Sigma _{11.2}$ is decomposed into the standard deviations of the residuals $\sigma _{x.z}$ and $\sigma _{y.z}$ after regressing *X* and *Y* on *Z*, and the partial correlation coefficient $\rho _{xy.z}$.

Under both null and alternative models, we set the following independent priors on the nuisance parameters $\theta _0$ (including the vector of means μ):

1 $$\begin{aligned} \begin{aligned} \mu&\propto 1, \textrm{B}\propto 1 \\ \Sigma _{22}&\propto |\Sigma _{22}|^{-(k + 1)/2} \\ \sigma _{x.z}&\propto \sigma _{x.z}^{\gamma -1}, \sigma _{y.z} \propto \sigma _{y.z}^{\delta -1}. \end{aligned} \end{aligned}$$

This choice of priors allows us to integrate nuisance parameters analytically. However, these priors are not chosen purely for this reason; they correspond to independent improper Jeffrey’s priors that are common for default Bayesian analyses, and ensure that the nuisance parameters do not affect the resulting Bayes factor. As will be discussed in Sect. 3.1, the resulting Bayes factor does depend on the hyperparameters δ and γ, but it is reasonable to typically set both to zero.

The alternative model includes one additional parameter, namely, the population partial correlation. The prior on $\rho _{xy.z}$ is set independently from the nuisance parameters as $\pi (\theta ) = \pi (\theta _0) \times \pi (\rho _{xy.z})$.

In accordance with the previous work on the Pearson’s correlation (Ly et al. 2018), the default prior used for the partial correlation here is a symmetric beta distribution stretched to the interval -1 to 1. This stretched beta prior has a single hyperparameter α that governs the concentration of the distribution around zero: if α=1, the prior distribution is uniform; when α is large, the distribution is peaked and centered on the value $\rho _{xy.z}=0$. Figure 1 shows four examples of the stretched beta prior distribution for $\rho _{xy.z}$ using different values for the α hyperparameter.

After integrating the nuisance parameters from both models, and integrating $\rho _{xy.z}$ from the alternative model, the Bayes factor in favor the alternative model compared to the null model is given by

2 $$\begin{aligned} \begin{aligned} \text {BF}_{10} = \frac{\mathcal {B}\left( \frac{1}{2}, \tilde{\alpha } \right) }{\mathcal {B}\left( \frac{1}{2}, \alpha \right) }\times _2F_1\left( \frac{n-k-\gamma -1}{2}, \frac{n-k-\delta -1}{2}; \tilde{\alpha } + \frac{1}{2}; r_{xy.z}^2\right) , \end{aligned} \end{aligned}$$

where $$\tilde{\alpha } = \alpha + \frac{n-k-\gamma -\delta -1}{2}$$.

The marginal posterior distribution of $\rho _{xy.z}$ under the alternative model is given by:

3 $$\begin{aligned} \begin{aligned}&\pi (\rho _{xy.z} \mid n, k, r_{xy.z}) \\&= \frac{(1-\rho _{xy.z}^2)^{(2\alpha + n - k - \gamma - \delta - 3)/2}}{\mathcal {B}\left( \frac{1}{2}, \alpha + \frac{n-k-\gamma -\delta -1}{2}\right) \phantom {.}_2F_1\left( \frac{n-k-\gamma -1}{2}, \frac{n-k-\delta -1}{2}; \alpha + \frac{n-k-\gamma -\delta }{2}; r_{xy.z}^2\right) } \\ \times&\left[ \phantom {.}_2F_1\left( \frac{n-k-\gamma -1}{2}, \frac{n-k-\delta -1}{2}; \frac{1}{2}; r_{xy.z}^2\rho _{xy.z}^2\right) + \right. \\&\left. 2r_{xy.z}\rho _{xy.z} W_{\gamma ,\delta }(n-k)\phantom {.}_2F_1\left( \frac{n-k-\gamma }{2}, \frac{n-k-\delta }{2}; \frac{3}{2}; r_{xy.z}^2\rho _{xy.z}^2\right) \right] . \end{aligned} \end{aligned}$$

Note that the expressions only depend on the data via the relevant summary statistic $r_{xy.z}$, the sample size n, and the number of conditioning variables k as promised. $\mathcal {B}(a,b)$ is the beta-function, $\phantom {.}_2F_1(a, b;c;z)$ is the Gaussian hypergeometric function, and $W_{\gamma , \delta } (\tilde{n})$ is defined by Ly et al. (2018, p. 7) as

4 $$\begin{aligned} \begin{aligned} W_{\gamma , \delta } (\tilde{n}) = \frac{\Gamma \left( \frac{\tilde{n}-\gamma }{2} \right) \Gamma \left( \frac{\tilde{n}-\delta }{2} \right) }{\Gamma \left( \frac{\tilde{n}-\gamma -1}{2} \right) \Gamma \left( \frac{\tilde{n}-\delta -1}{2} \right) }. \end{aligned} \end{aligned}$$

By substituting $\tilde{n} = n-k$ in Eq. 3, the number of samples exceeding the number of controlling variables, we see that the results generalize the Pearson’s correlation of Ly et al. (2018) in the sense that the Pearson’s correlation is a special case of the partial correlation when the number of controlling variables is zero (k=0).

## Properties of the Bayes factor

Bayesian model comparison and selection methods, such as the Bayes factor, are sensitive to the choice of priors, and this sensitivity does not vanish as the sample size increases (Kass and Raftery 1995; Bayarri et al. 2012).

Because of this sensitivity, a considerable effort has been exerted to develop “objective”, or “default” methods that would provide standard inferences for typical testing scenarios Berger (2006); Bayarri et al. (2012). A pioneer in this field was Jeffreys (1961) who not only proposed various default Bayesian tests for common statistical problems, but also provided a set of *desiderata* for newly developed tests, such that they provide an intuitive framework for inference (Ly et al. 2016b, a; Bayarri et al. 2012).

Here we discuss that the Bayes factor presented in this article meets Jeffreys’s desiderata for Bayes factors: predictive matching, information consistency, and model selection consistency.

### Predictive matching

A Bayes factor is predictively matched if it equals 1 when the Bayes factor is presented with completely uninformative data, that is, when the data bear no evidence for either hypothesis.

This occurs when the data are of insufficient size, thus, less than a minimal sample size needed to distinguish between the null and alternative models. For these data sets the Bayes factor should remain indifferent (i.e., $\text {BF} = 1$).

Note that we cannot infer the partial correlation when n≤k+1 as the sample partial correlation is then undefined. When n=k+2, we automatically get $r_{xy.z} = \pm 1$, regardless of the value of the population coefficient. Hence, the minimum sample size is $n_{\min } = k+3$. For data sets of size n<k+1, we define the Bayes factor to be one. For n=k+2, we enter the values $r_{xy.z} = 1$ into Eq. (2) and obtain with $\tilde{\alpha } = \alpha + \frac{n-k-\gamma -\delta -1}{2}$

5 $$\begin{aligned} \text {BF}_{10}&= \frac{\mathcal {B}\left( \frac{1}{2}, \tilde{\alpha } \right) }{\mathcal {B}\left( \frac{1}{2}, \alpha \right) }\times _2F_1\left( \frac{1-\gamma }{2}, \frac{1-\delta }{2}; \tilde{\alpha } + \frac{1}{2}; 1\right) \nonumber \\&= \frac{\mathcal {B}\left( \frac{1}{2}, \tilde{\alpha } \right) }{\mathcal {B}\left( \frac{1}{2}, \alpha \right) }\times \frac{\Gamma (\tilde{\alpha } + \frac{1}{2})\Gamma (\tilde{\alpha } + \frac{1}{2} - \frac{1-\gamma }{2} - \frac{1-\delta }{2})}{\Gamma (\tilde{\alpha }+\frac{1}{2}-\frac{1-\gamma }{2})\Gamma (\tilde{\alpha }+\frac{1}{2}-\frac{1-\delta }{2})} \nonumber \\&= \frac{\Gamma (\alpha + \frac{1-\gamma -\delta }{2})\Gamma (\alpha + \frac{1}{2})}{\Gamma (\alpha + \frac{1-\gamma }{2})\Gamma (\alpha + \frac{1-\delta }{2})}. \end{aligned}$$

Thus, for the Bayes factor to be predictively matched, we require $\gamma = \delta = 0$.

### Information consistency

A Bayes factor is information consistent if it diverges to infinity, thus, falsifies the null, when the Bayes factor is presented with overwhelmingly informative data.

Overwhelmingly informative data sets are of sufficient size, thus, $n \ge n_{\min } = k + 3$ with $r_{xy.z} = 1$ or $r_{xy.z} = -1$, because if the null were true, then such an event occurs with chance zero. With $r_{xy.z} = \pm 1$ the Bayes factor can be simplified as

6 $$\begin{aligned} \text {BF}_{10} = \frac{2^{1-2 \alpha } \sqrt{ \pi } }{\mathcal {B}(\alpha , \alpha )} \frac{ \Gamma ( \alpha + \tfrac{ \tilde{n} -1}{2}) \Gamma ( \alpha + \tfrac{ \tilde{n} }{2}) \Gamma ( \alpha + 1- \tfrac{\tilde{n}}{2}) }{\Gamma ( \alpha + \tfrac{\tilde{n}}{2}) \Gamma ( \alpha + \tfrac{1}{2}) \Gamma ( \alpha + \tfrac{1}{2})}, \end{aligned}$$

with $\tilde{n} \ge n - k$. This Bayes factor diverges when one of the gamma functions in the numerator has a non-positive argument, thus, when $\alpha + 1 - \tfrac{ \tilde{n}}{2} \le 0$, hence, when $2 \alpha + 2 + k \le n$. For this to already occur at $n = n_{\min }$ we require $\alpha \le 1/2$.

On the other hand, when $r_{xy.z} = \pm 1$ and α>0 is given, we conclude that the Bayes factor diverges whenever $n - k \ge 2 \alpha + 2$. For instance, for α=1 the Bayes factor diverges only when n≥k+4, thus, missing the information consistency desideratum by one observation. In other words, for α=1, we are one observation too reluctant to falsify the null and without that extra observation (that is, with $n=n_\text {min}$), $\text {BF}_{10}$ is bounded at 2. Analogously, for α=20 we require an additional 39 perfectly (partially) correlated observations before we are willing to falsify the null, and at $n=n_\text {min}$ the maximum possible $\text {BF}_{10}$ is merely ≈1.02. Choosing α larger than $\frac{1}{2}$ can therefore yield “severely” inconsistent Bayes factor (the limiting bound of the BF is close to 1 but points in the direction of the correct model; Mulder et al. 2021), which can be an issue mainly with small sample sizes. On the other hand, the consistency bound $n - k \ge 2 \alpha + 2$ is relatively forgiving, meaning that the null can be in principle falsified with modest sample sizes.

### Model selection consistency

Model selection consistency of the Bayes factor refers to the asymptotic property that, as the sample size grows, the Bayes factor increasingly favors the true data-generating model over the competing (incorrect) model (see Chib & Kuffner, 2016).

We briefly sketch the argument for model selection consistency of the proposed Bayes factor, which closely parallels the corresponding argument for the model selection consistency of a Bayes factor for a Pearson correlation coefficient by Ly et al. (2016b).

Under standard regularity conditions for the Gaussian model, the sample partial correlation $r_{xy.z}$ is asymptotically normal with mean $\rho _{xy.z}$ and variance $\frac{1}{(n-k)(1-\rho _{xy.z}^2)^2}$. Under the null hypothesis $\rho _{xy.z}=0$, we have $r_{xy.z}^2 = O_p\!\left( (n-k)^{-1}\right) .$

The Bayes factor $\text {BF}_{10}$ depends on the data only through $r_{xy.z}^2$ and is an analytic function of $r_{xy.z}^2$ in a neighborhood of zero. A Taylor expansion of the hypergeometric term in $\text {BF}_{10}$ around $r_{xy\cdot z}^2 = 0$ implies that this term remains stochastically bounded as $n\rightarrow \infty$. Meanwhile, the beta-function prefactor in $\text {BF}_{10}$ decreases at rate $(n-k)^{-1/2}$. Thus, under the null model, the Bayes factor approaches zero as *n* increases.

Under the alternative hypothesis $\rho _{xy.z}\ne 0$, consistency of the sample partial correlation implies $r_{xy.z}\rightarrow \rho _{xy.z}$ in probability. For such values of $r_{xy.z}^2$, the hypergeometric function appearing in $\text {BF}_{10}$ increases rapidly as the effective sample size n-k grows. This reflects the fact that the likelihood under the alternative model concentrates around the true nonzero partial correlation, while the null model evaluates the likelihood at the fixed, incorrect value $\rho _{xy.z}=0$. As a result, under the alternative model $\text {BF}_{10}$ diverges to infinity as sample size grows indefinitely.

**Fig. 2:**
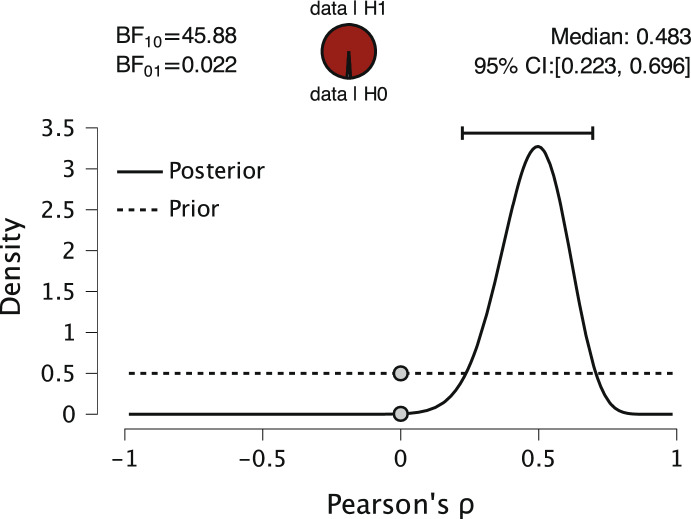
Bayesian inference on the Pearson’s correlation coefficient Ly et al. 2018) for the data reported in Lleras et al. (2011) (i.e., r=.51, n=40). The Bayes factor indicates very strong support for the alternative model over the null model. The gray dots at ρ=0 visualize the Savage-Dickey density ratio (Dickey and Lientz 1970). Figure concept from JASP (e.g., van Doorn et al. 2021)

**Fig. 3:**
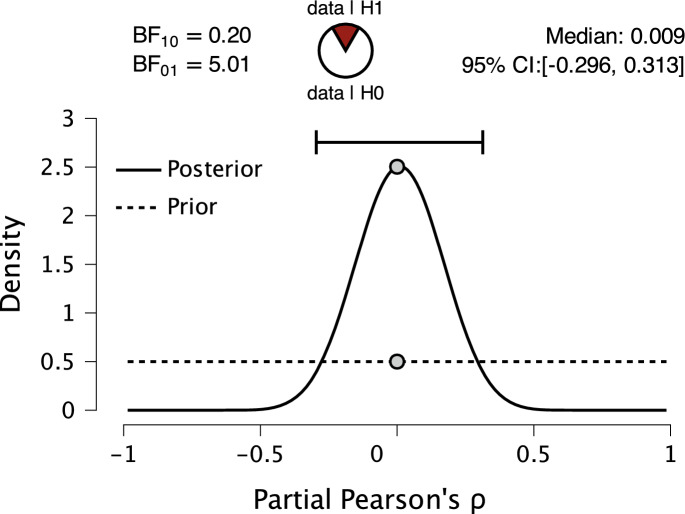
Bayesian inference on the partial correlation coefficient for the data reported in Lleras et al. (2011) (i.e., $r_{xy.z}=.01$, n=40). After controlling for participant age, the Bayes factor indicates moderate support for the null model over the alternative model. The gray dots at ρ=0 visualize the Savage-Dickey density ratio (Dickey and Lientz 1970). Figure concept from JASP (e.g., van Doorn et al. 2021)

**Fig. 4:**
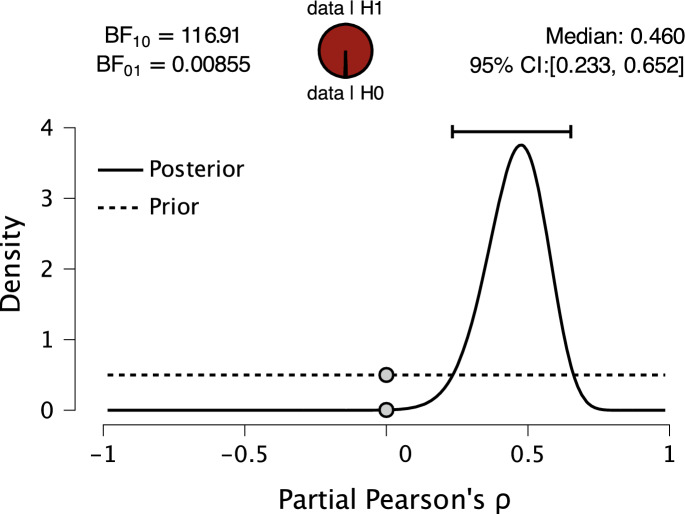
Bayesian inference on the partial correlation coefficient for the data reported in Coccia (2021). The Bayes factor indicates extreme support for the alternative model over the null model. The grey dots at ρ=0 visualize the Savage-Dickey density ratio (Dickey and Lientz 1970). Figure concept from JASP (e.g., van Doorn et al. 2021)

**Fig. 5:**
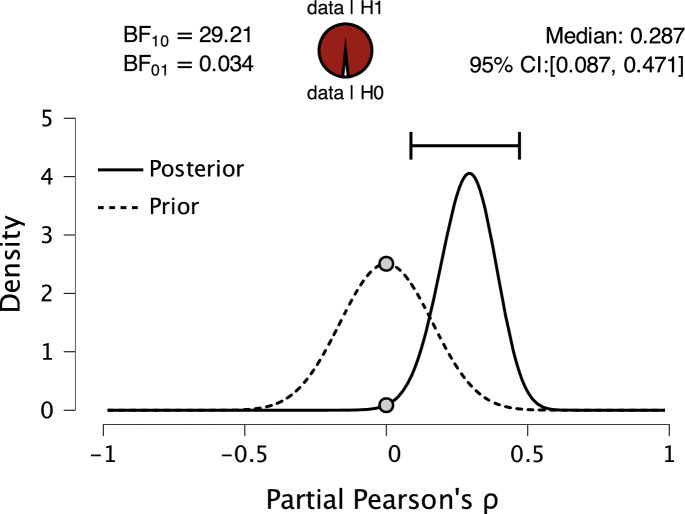
Bayesian inference on the partial correlation coefficient for the data reported in Coccia (2021), using the informed prior with α=20. Compared to the results based on the uniform prior, the posterior distribution has shifted toward zero, and the Bayes factor is less compelling; however, the support for the alternative model over the null model is still strong. The gray dots at ρ=0 visualize the Savage-Dickey density ratio (Dickey and Lientz 1970). Figure concept from JASP (e.g., van Doorn et al. 2021)

**Fig. 6:**
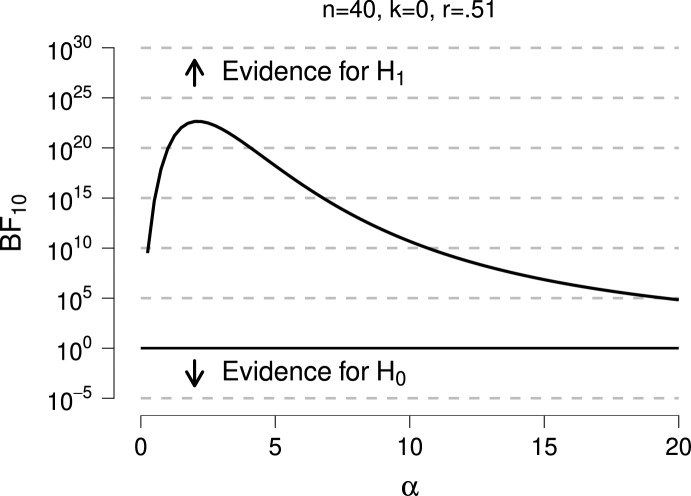
Bayes factor robustness plot (see, van Doorn et al. 2021) for the correlation reported by Lleras et al. (2011): Bayes factor is plotted as a function of the prior width α between 0.25 and 20. The evidence in favor of the alternative model is strong for a wide range of prior specifications

**Fig. 7:**
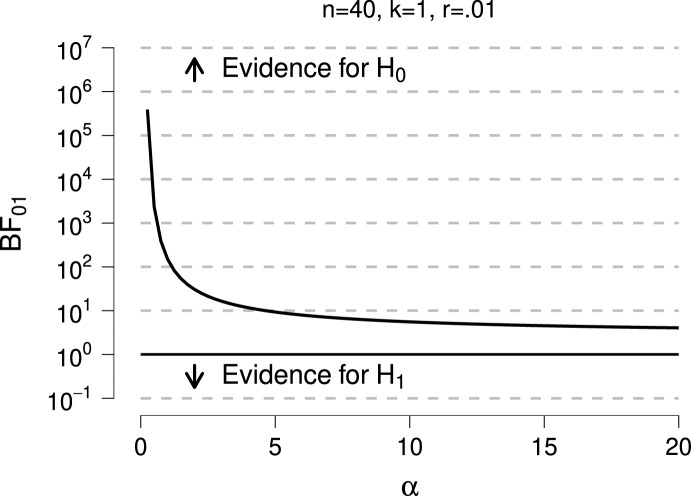
Bayes factor robustness plot (see, van Doorn et al. 2021) for the partial correlation reported by Lleras et al. (2011): Bayes factor is plotted as a function of the prior width α between 0.25 and 20. The evidence favors the null model especially for small values of α. Larger α represent priors concentrated around the null value ρ=0 and the evidence quickly decreases

**Fig. 8:**
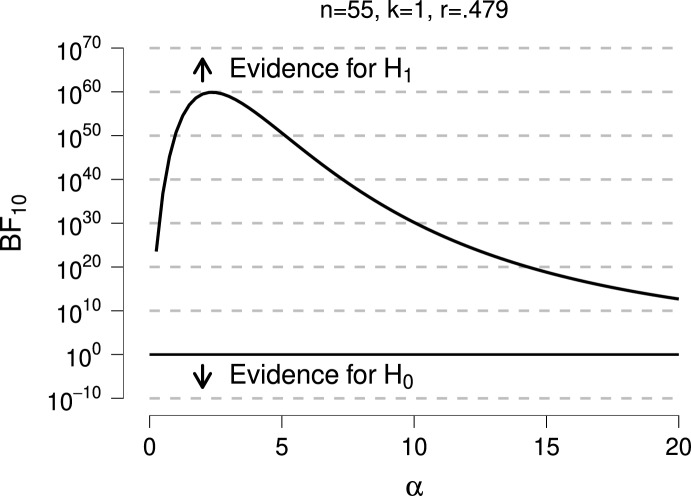
Bayes factor robustness plot (see, van Doorn et al. 2021) for the partial correlation reported by Coccia (2021): Bayes factor is plotted as a function of the prior width α between 0.25 and 20. The evidence in favor of the alternative model is strong for a wide range of prior specifications

This is in line with a general intuition for nested hypotheses provided by Jeffreys (1935, 1936), whereby approximating the Bayes factor (in favor of the null) using the assumption that the sampling distribution of the MLE ($r_{xy.z}$) is approximately normal yields an expression dependent on factor of $\sqrt{n}$ and a negative squared exponential factor of the distance of the MLE from the test value, relative to the standard error (Wagenmakers 2022). Under the null model, the term $\sqrt{n}$ dominates and the support for null diverges to infinity, whereas under the alternative, the evidence in support of null goes to zero, resulting in model selection consistency (Velidi et al. 2025).

## Two examples

Our first example features a data set from experimental psychology. Lleras et al. 2011 had n=40 participants complete a visual search task. The data showed a relatively high correlation (r=.51) between ‘successful search time’ and ‘rapid resumption’. In a Bayesian reanalysis, we assigned the correlation coefficient a stretched beta prior with α=1 (uniform prior between -1 and 1). Figure 2 shows the resulting inference. In terms of testing, the Bayes factor $\text {BF}_{10} \approx 45.88$ indicates ‘very strong’ evidence in favor of the alternative model over the null model (cf. Jeffreys 1961, Appendix B; (Lee and Wagenmakers 2013), Table 7.1). In terms of estimation, the posterior distribution for Pearson’s ρ (under the alternative model) is relatively symmetric around 0.483, with a central 95% credible interval that ranges from 0.223 to 0.696.

However, after controlling for participant’s age the sample partial correlation coefficient decreases to almost zero ($r_{xy.z} =.01$). In a Bayesian reanalysis, we again assigned the partial correlation coefficient a stretched beta distribution with α=1 (uniform prior between -1 and 1). Figure 3 shows the resulting inference. In terms of testing, the Bayes factor $\text {BF}_{01} \approx 5.01$ indicates ‘moderate’ evidence in favor of the null model over the alternative model (cf. Jeffreys 1961, Appendix B; Lee and Wagenmakers 2013, Table 7.1). For comparison, Wetzels and Wagenmakers (2012) reported $\text {BF}_{01} = 7.70$ and Wang et al. (2019) reported $\text {BF}_{01} = 2.02$. The method by Williams and Mulder (2020) requires the full sample covariance matrix of the three variables (‘successful search time’, ‘rapid resumption’, and ‘age’) to obtain the Bayes factor for the partial correlation. To our knowledge, this information is unfortunately not openly available.

In terms of estimation, the posterior distribution for $\rho _{xy.z}$ (under the alternative model) is relatively symmetric around 0.009, with a central 95% credible that ranges from -0.296 to 0.313.

Our second example concerns the relation between COVID-19 infections and air pollution across 55 Italian cities in the period from March 17^th^ to April 7^th^, 2020. Specifically, Coccia (2021) reported a partial correlation between the logarithm of COVID-19 infections and the logarithm of the number of days with increased air pollution, controlling for population density. The summary statistics that allow a complete Bayesian reanalysis are the sample partial correlation $r_{xy.z} =.479$, sample size n=55, and the number of controlling variables (population density) k=1. We again assigned the partial correlation coefficient a stretched beta prior with α=1 (uniform between -1 and 1). Figure 4 shows the resulting inference. In terms of testing, the Bayes factor $\text {BF}_{10} \approx 116.91$ indicates ‘extreme’ evidence in favor of the alternative model over the null model (cf. Jeffreys 1961, Appendix B; Lee and Wagenmakers 2013, Table 7.1). In terms of estimation, the posterior distribution for $\rho _{xy.z}$ (under the alternative model) is relatively symmetric around 0.460, with a central 95% credible that ranges from 0.233 to 0.652. The data therefore appear to provide compelling statistical support for an association between the intensity of air pollution and the susceptibility to COVID-19 infections.

To explore the robustness of this conclusion, we may reanalyze the data from Coccia (2021) using an informed prior that assigns more mass to values of $\rho _{xy.z}$ near zero. Specifically, we assign $\rho _{xy.z}$ a stretched beta distribution with the α hyperparameter set to 20. The result is shown in Fig. 5. Compared to the result with a uniform prior, the posterior distribution is now closer to zero, and Bayes factor is noticeably smaller: $\text {BF}_{10} \approx 29.21$. However, the data still provide strong evidence in favor of the alternative model over the null model.

Instead of picking a single value of the hyperparameter α to explore the robustness of the result, we may report the Bayes factor for a range of possible prior specifications (see, van Doorn et al. 2021). Robustness plots in Figs. 6, 7 and 8 show Bayes factors for the three coefficients for a range of different values of α between 0.25 and 20. Table 2 further shows the numerical values of the Bayes factor for values of $\alpha \in \{\frac{1}{2}, 1, 5, 20\}$ (these particular priors are displayed in Fig. 1). Generally, with increasing α, the prior under the alternative becomes more concentrated around zero, and the predictions from the alternative model become similar to the null model. Therefore, the Bayes factor tends to decrease toward indecision. In the correlation example from Lleras et al. (2011) (Fig. 7) and the partial correlation example from Coccia (2021) (Fig. 8), the evidence in favor of the alternative model remains strong even for large values of α. For the partial correlation example from Lleras et al. (2011) where the data favor the null model (Fig. 6), the evidence is less conclusive; for small values of $\alpha =\frac{1}{2}$, the Bayes factor shows moderate evidence against the alternative $\text {BF}_{10}=0.129$, but the strength of the evidence rapidly declines with increasing α.

## Conclusion & discussion

We presented a new approach for Bayesian testing and estimation of a partial correlation coefficient. The framework generalizes previous work on Pearson’s correlation coefficient (Ly et al. 2016b, 2018) and inherits several desirable properties; for instance, when $\delta = \gamma = 0$ the Bayes factor is predictively matched, and when $\alpha \le 1/2$ also information consistent. The Bayes factor is also model selection consistent. The inference is carried out on the partial correlation coefficient itself, as opposed to two previous proposals of Bayesian tests of partial correlations. As a result, we obtained an analytic expression for the Bayes factor and for the posterior distribution of the partial correlation coefficient. Furthermore, the full inference can be carried out using only the sample size, the number of controlling variables, and the relevant summary statistics, that is, only the sample partial correlation $r_{xy.z}$ corresponding to the target of inference $\rho _{xy.z}$. For these reasons, the methodology developed here is arguably an attractive option as a default Bayesian inference procedure for partial correlations.

**Table 2:** Sensitivity of the Bayes factor toward prior specification. The table shows the Bayes factor against the null hypothesis with different width of the stretched beta prior shown in Fig. 1

				$$\text {BF}_{10}$$			
Study	r	n	k	$\alpha =\frac{1}{2}$	α=1	α=5	α=20
Lleras et al. (2011)	.51	40	0	33.85	45.88	41.94	11.13
Lleras et al. (2011)	.01	40	1	0.129	0.20	0.44	0.71
Coccia (2021)	.479	55	1	84.70	116.91	116.51	29.21

The proposed Bayes factor and posterior density require evaluating the Gaussian hypergeometric function, which is known to be prone to numerical instabilities. In the present paper, we relied on its implementation of Hankin (2015) which provided stable results for a reasonable range of statistics. However, in extreme settings, overflow or loss of precision may still arise. In these cases, approximating the function might be necessary in order to obtain numerically stable results (Butler and Wood 2002).

It is important to note that the Bayesian and frequentist inference for a partial correlation share many key assumptions: the vector of observations must be independent, and the variables must be (approximately) multivariate-normally distributed. Although small deviations from these assumptions may not completely invalidate the results, the coefficient may be especially sensitive to distortion in case of nonlinear relationships between variables, in the presence of outliers, or with significant measurement error (Osborne and Waters 2002; Vargha et al. 2013; Liu 1988; Quade 2017). Whenever researchers have access to the raw data, we recommend that they carefully check these assumptions; when researchers report original work we encourage them to publicly archive the (properly anonymized) data, or—at a minimum—plot the data so that readers may confirm that the analysis is appropriate and informative (e.g., Wagenmakers et al. 2021).

## Appendix: Derivation of the main results

### Reparametrizing the multivariate normal model

Let $X\in \mathbb {R}$, $Y\in \mathbb {R}$ be two random variables and $Z\in \mathbb {R}^k$ a random vector ($k\in \mathbb {Z}_{\ge 0}$). We consider the random vector $W\in \mathbb {R}^p$ collecting *X*, *Y*, *Z*, where p=k+2, to follow the multivariate normal distribution. Further, let *n* be the number of observations of the random vector *W*, and summarize the observed data as follows: $d = (n, \bar{w}, S)$, where $\bar{w}$ is the vector of sample means, $\bar{w}^{(j)} = n^{-1} \sum _i^n w_i^{(j)}$ for $j \in {1,...,p}$, and $S = n^{-1} (w-\bar{w})'(w-\bar{w})$ is an average sum of squares and cross-product matrix.

The likelihood of the model is

7 $$\begin{aligned} f(d \mid \mu , \Sigma ) = (2\pi )^{-\frac{pn}{2}} |\Sigma |^{-\frac{n}{2}} \exp \left( -\frac{n}{2} [\text {tr}(\Sigma ^{-1}S) + (\bar{w}-\mu )'\Sigma ^{-1}(\bar{w}-\mu )]\right) , \end{aligned}$$

where $\text {tr}(.)$ denotes the trace operator and $|\phantom {.}.\phantom {.}|$ denotes the determinant, μ is the vector of population means, and Σ is the population variance-covariance matrix.

As there are *p* population means, *p* variances, and ${p\atopwithdelims ()2}$ correlations, the model contains p(p+3)/2 parameters. We will remove the nuisance parameters by carefully setting suitable prior distributions and marginalizing them out of the likelihood. Specifically, we choose priors such that the nuisance parameters do not influence the inference about $\rho _{xy.z}$. This can be accomplished by allowing the structure of the parameters (and their priors) to reflect the structure of the likelihood.

Our approach is to reparametrize the multivariate normal model of the random vector *W* in order to isolate the partial correlation $\rho _{xy.z}$ as a separate parameter. It is then possible to fix the value of $\rho _{xy.z}$ to zero (under the null model $\mathcal {M}_0$), or assign it a prior probability distribution (under the alternative model $\mathcal {M}_1$). The desired reparametrization is achieved using the Schur decomposition of the variance-covariance matrix, which replaces Σ with three sets of parameters: $\Sigma _{11.2}$, $\Sigma _{22}$, and $\textrm{B}$. First, $\Sigma _{11.2}$ is the conditional variance-covariance matrix of the variables *X* and *Y* after controlling for variables *Z*. $\Sigma _{11.2}$ can be decomposed in terms of three parameters: the focal parameter $\rho _{xy.z}$ that represents the partial correlation, and the two standard deviations of the residuals, $\sigma _{x.z}$ and $\sigma _{y.z}$, after regressing *X* and *Y* on *Z*, respectively. Second, $\Sigma _{22}$ is the variance-covariance matrix of the controlling variables *Z*; third, $\textrm{B}$ is a matrix of regression coefficients that represent the relation of *X* and *Y* to *Z*.

We achieve the reparametrization by first decomposing Σ into a block matrix,

8 $$\begin{aligned} \Sigma = \begin{bmatrix} \Sigma _{11} & \quad \Sigma _{12} \\ \Sigma _{12} & \quad \Sigma _{22} \end{bmatrix}, \end{aligned}$$

where $\Sigma _{11}$ is a 2×2 variance-covariance matrix of the variables *X* and *Y*, $\Sigma _{22}$ is a k×k variance-covariance matrix of the controlling variables *Z*, and $\Sigma _{12} = \Sigma _{21}'$ is a 2×k matrix of cross-covariances of *X* with *Z*, and *Y* with *Z*.

To obtain the partial correlation instead of the classic correlation, we use the Schur complement of $\Sigma _{11}$ in Σ defined as $\Sigma _{11.2}~=~\Sigma _{11}-\Sigma _{12}\Sigma _{22}^{-1}\Sigma _{21}$, which for the multivariate normal model can be interpreted as the conditional variance-covariance matrix of *X* and *Y* given *Z*. This can be done analogously with the sample counterpart *S* provided that $S_{22}$ is positive definite.

We now need to rewrite the trace in the exponent of the likelihood in Eq. (13) in terms of this decomposition. The Schur decomposition allows us to invert Σ easily as:

$$\begin{aligned} \Sigma ^{-1} = \begin{bmatrix} \Sigma _{11.2}^{-1} & \quad -\Sigma _{11.2}^{-1}\Sigma _{12}\Sigma _{22}^{-1} \\ & \\ -\Sigma _{22}^{-1}\Sigma _{21}\Sigma _{11.2}^{-1} & \quad \Sigma _{22}^{-1} + \Sigma _{22}^{-1}\Sigma _{21}\Sigma _{11.2}^{-1}\Sigma _{12}\Sigma _{22}^{-1} \end{bmatrix}. \end{aligned}$$

To let the sample partial covariance appear in the likelihood, we write *S* as a block matrix:

$$\begin{aligned} S = \begin{bmatrix} S_{11.2} + S_{12}S_{22}^{-1}S_{21} & S_{12} \\ S_{21} & S_{22} \end{bmatrix}. \end{aligned}$$

This leads to

9 $$\begin{aligned} \begin{aligned} \text {tr}(\Sigma ^{-1}S)&= \text {tr}(\Sigma _{11.2}^{-1}S_{11.2}) + \text {tr}(\Sigma _{22}S_{22}) \\&\quad + \text {tr}(\Sigma _{11.2}^{-1}S_{12}S_{22}^{-1}S_{21}) - \text {tr}(\Sigma _{11.2}^{-1}\Sigma _{12}\Sigma _{22}^{-1}S_{21}) \\&\quad - \text {tr}(\Sigma _{22}^{-1}\Sigma _{21}\Sigma _{11.2}^{-1}S_{12}) + \text {tr}(\Sigma _{22}^{-1}\Sigma _{21}\Sigma _{11.2}^{-1}\Sigma _{12}\Sigma _{22}^{-1}S_{22}). \end{aligned} \end{aligned}$$

The first trace involves the block of interest (i.e., the partial correlation scaled by the partial variance) and the second trace involves the nuisance covariance of *Z*. The last four terms involve the nuisance blocks with respect to the cross-correlation $\Sigma _{12}$, and can be written (by factoring out $S_{22}$ and $\Sigma _{11.2}$) as a quadratic term in the form

$$\begin{aligned} \text {tr}\left[ S_{22}(S_{12}S_{22}^{-1} - \Sigma _{12}\Sigma _{22}^{-1})'\Sigma _{11.2}^{-1}(S_{12}S_{22}^{-1} - \Sigma _{12}\Sigma _{22}^{-1})\right] , \end{aligned}$$

where we used the cyclic property and linearity of traces, and multiplied by the identity $I = S_{22}S_{22}^{-1}$ where needed in order to collect the four traces inside of a single quadratic term, as detailed in the Appendix B. Substituting the four traces with the quadratic terms leads to

10 $$\begin{aligned} \begin{aligned} \text {tr}(S\Sigma ^{-1})&= \text {tr}(S_{11.2}\Sigma _{11.2}^{-1}) + \text {tr}(S_{22}\Sigma _{22}^{-1}) \\&\quad + \text {tr}\left[ S_{22}(S_{12}S_{22}^{-1} - \Sigma _{12}\Sigma _{22}^{-1})'\Sigma _{11.2}^{-1}(S_{12}S_{22}^{-1} - \Sigma _{12}\Sigma _{22}^{-1})\right] . \end{aligned} \end{aligned}$$

In the above equation, the nuisance block including the terms $\Sigma _{12}\Sigma _{22}^{-1}$ can be interpreted as the linear regression coefficients of *X* and *Y* on *Z*. We write the matrix as $\textrm{B}= \Sigma _{12}\Sigma _{22}^{-1}$ and analogously $\hat{\textrm{B}} = S_{12}S_{22}^{-1}$. Using the fact that $|\Sigma | = | \Sigma _{11.2}| \times |\Sigma _{22}|$, we arrive at

11 $$\begin{aligned} \begin{aligned} f(d \mid \mu , \Sigma )&= (2\pi )^{-\frac{pn}{2}} |\Sigma _{11.2}|^{-\frac{n}{2}} |\Sigma _{22}|^{-\frac{n}{2}}\\ &\quad \times \exp \Big (-\frac{n}{2}(\bar{w}-\mu )'\Sigma ^{-1}(\bar{w}-\mu )\Big ) \\ &\quad \times \exp \Big (-\frac{n}{2}\text {tr}(S_{11.2}\Sigma _{11.2}^{-1})\Big ) \\&\quad \times \exp \Big (-\frac{n}{2}\text {tr}(S_{22}\Sigma _{22}^{-1})\Big ) \\&\quad \times \exp \Big (-\frac{n}{2}\text {tr}\left[ S_{22}(\hat{\textrm{B}} - \textrm{B})'\Sigma _{11.2}^{-1}(\hat{\textrm{B}} - \textrm{B})\right] \Big ). \end{aligned} \end{aligned}$$

Equation (11) shows that the multivariate normal model can be reparametrized such that the sets of parameters μ, $\Sigma _{11.2}$, $\Sigma _{22}$, and $\textrm{B}$ can be integrated out independently of one another, if the prior factorizes similarly.

### Integrating out the nuisance parameters

Equation (11) shows that the multivariate normal likelihood factorizes in four parts, one that involves the parameter of interest, one that involves the nuisance block regarding the variance-covariance of the conditioning variables *Z*, one that involves the regression of *X* and *Y* on *Z*, and the vector of means. This decomposition suggests prior distributions of the form $\pi (\mu , \Sigma ) = \pi (\mu )\pi (\textrm{B})\pi (\Sigma _{22})\pi (\Sigma _{11.2})$. Because μ and $\textrm{B}$ act as location parameters and $\Sigma _{22}$ represents a scaling factor, we choose the following priors: $\mu \propto 1$, $\textrm{B}\propto 1$, and $\Sigma _{22} \propto |\Sigma _{22}|^{-(k + 1)/2}$. Note that improper priors generally do not cause complications for the computation of Bayes factors, with the provisos that the joint posterior distribution is proper, and that the improper priors are assigned exclusively to nuisance parameters and emphatically not to the test-relevant parameter (cf. Ly et al. 2016a; Wagenmakers and Ly 2023).

#### Integrating out μ

With improper priors $\mu _i \propto 1$ for $i \in {1,...,p}$ we can integrate μ out of the likelihood. This leads to a simple Gaussian integral and costs one degree of freedom:

12 $$\begin{aligned} \begin{aligned} \int \exp \left( -\frac{1}{2} (\bar{w}-\mu )'(n^{-1}\Sigma )^{-1}(\bar{w}-\mu )\right) ~d\mu = |2\pi n^{-1}\Sigma |^{1/2} = (2\pi n^{-1})^{p/2} |\Sigma |^{1/2}. \end{aligned} \end{aligned}$$

The resulting reduced likelihood is

13 $$\begin{aligned} f(d \mid \Sigma _{11.2}, \Sigma , \textrm{B})= &  (2\pi )^{p(1-n)/2} n^{-p/2} |\Sigma _{11.2}|^{(1-n)/2} |\Sigma _{22}|^{(1-n)/2}\nonumber \\  &  \times \exp \Big (-\frac{n}{2}\text {tr}(S_{11.2}\Sigma _{11.2}^{-1})\Big ) \nonumber \\ &  \times \exp \Big (-\frac{n}{2}\text {tr}(S_{22}\Sigma _{22}^{-1})\Big ) \nonumber \\ &  \times \exp \Big (-\frac{n}{2}\text {tr}\left[ S_{22}(\hat{\textrm{B}} - \textrm{B})'\Sigma _{11.2}^{-1}(\hat{\textrm{B}} - \textrm{B})\right] \Big ), \end{aligned}$$

leaving us with p(p+1)/2 parameters.

#### Integrating out $\textrm{B}$

To integrate out $\textrm{B}$, observe that the relevant part of the likelihood is a kernel of a 2×k matrix normal distribution with a location matrix $\textrm{B}\in \mathbb {R}^{2\times k}$, one 2×2 scale matrix $\Sigma _{11.2}$, and a second, k×k, scale matrix $S_{22}$. By assigning priors on $\textrm{B}_{ij} \propto 1$ for i∈1,2, and j∈1,...,k, we get:

14 $$\begin{aligned} \begin{aligned}&\int \exp \left( -\frac{n}{2} \text {tr}\left[ S_{22}(\hat{\textrm{B}} - \textrm{B})'\Sigma _{11.2}^{-1}(\hat{\textrm{B}} - \textrm{B})\right] \right) d\textrm{B}= (2\pi )^{k} n^{-k} |S_{22}|^{-1} |\Sigma _{11.2}|^{k/2}, \end{aligned} \end{aligned}$$

provided that $|S_{22}| > 0$ and $|\Sigma _{11.2}| > 0$. The resulting reduced likelihood is

15 $$\begin{aligned} \begin{aligned} f(d \mid \Sigma _{11.2}, \Sigma )&= |S_{22}|^{-1} (2\pi )^{p(1-n)/2 + k} n^{-p/2 - k} |\Sigma _{11.2}|^{(1-n+k)/2} |\Sigma _{22}|^{(1-n)/2} \\ &\quad \times \exp \Big (-\frac{n}{2}\text {tr}(S_{11.2}\Sigma _{11.2}^{-1})\Big ) \\&\quad \times \exp \Big (-\frac{n}{2}\text {tr}(S_{22}\Sigma _{22}^{-1})\Big ), \end{aligned} \end{aligned}$$

leaving us with 3+k(k+1)/2 parameters.

#### Integrating out $\Sigma _{22}$

To integrate out $\Sigma _{22}$, we collect the relevant part of the likelihood and the prior and observe that it results in a Wishart integral:

16 $$\begin{aligned} \begin{aligned}&\int |\Sigma _{22}|^{-(k+1)/2} |\Sigma _{22}|^{(1-n)/2} \exp \left( -\frac{n}{2}\text {tr}(S_{22}\Sigma _{22}^{-1})\right) d\Sigma _{22} \\&\quad =\int |\Sigma _{22}|^{-(n+k)/2} \exp \left( -\frac{n}{2}\text {tr}(S_{22}\Sigma _{22}^{-1})\right) d\Sigma _{22} \\&\quad =2^{(n+k)k/2}\times \Gamma _k\Big (\frac{n+k}{2}\Big )\times |S_{22}|^{-(n-1)/2}, \end{aligned} \end{aligned}$$

where $\Gamma _k(.)$ is the *k*-variate gamma function. The resulting reduced likelihood is

17 $$\begin{aligned} f(d \mid \Sigma _{11.2})= &  \overbrace{2^{(n+k)k/2}\times \Gamma _k\Big (\frac{n+k}{2}\Big )|S_{22}|^{-(1+n)/2} (2\pi )^{p(1-n)/2 + k} n^{-p/2 - k}}^{C(n, k, S_{22})} \nonumber \\  &  \times |\Sigma _{11.2}|^{(1-n+k)/2} \exp \Big (-\frac{n}{2}\text {tr}(S_{11.2}\Sigma _{11.2}^{-1})\Big ), \end{aligned}$$

leaving us with just three parameters.

### Final derivation of the bayes factor and the marginal posterior distribution

The resulting reduced likelihood contains only three parameters: the partial correlation $\rho _{xy.z}$ and the residual variances $\sigma _{x.z}^2$ and $\sigma _{y.z}^2$. Expressing the reduced likelihood Eq. (17) in terms of these quantities yields

$$\begin{aligned} \begin{aligned}&f(s_{x.z}, s_{y.z}, r_{xy.z} \mid \sigma _{x.z}, \sigma _{y.z}, \rho _{xy.z}) \\ &= C(n, k, S_{22}) \\ &\times \left( \sqrt{1-\rho _{xy.z}^2}\sigma _{x.z}\sigma _{y.z}\right) ^{1-n+k} \\&\times \exp \left( {-\frac{n}{2(1-\rho _{xy.z}^2)}}\left[ \frac{s_{x.z}^2}{\sigma _{x.z}^2} - 2\rho _{xy.z}\frac{r_{xy.z}s_{x.z}s_{y.z}}{\sigma _{x.z}\sigma _{y.z}} + \frac{s_{y.z}^2}{\sigma _{y.z}^2}\right] \right) . \end{aligned} \end{aligned}$$

which is similar to the likelihood for the Pearson’s correlation coefficient described in (Ly et al. 2018, Eq.8), the only difference being the additional *k* term in the exponent of the determinant. This allows us to use of Ly et al. (2018). In sum, if we use the prior

18 $$\begin{aligned} \mu \propto 1, \textrm{B}\propto 1, \Sigma _{22} \propto |\Sigma _{22}|^{-(k + 1)/2}, \end{aligned}$$

19 $$\begin{aligned} \sigma _{x.z} \propto \sigma _{x.z}^{\gamma -1}, \sigma _{y.z} \propto \sigma _{y.z}^{\delta -1}, \text { and } \end{aligned}$$

20 $$\begin{aligned} \rho \sim \text {stretched beta}(\alpha , \alpha ) \end{aligned}$$

we then obtain the Bayes factor for the alternative model over the null model is given by

21 $$\begin{aligned} \begin{aligned} \text {BF}_{10} = \frac{\mathcal {B}\left( \frac{1}{2}, \tilde{\alpha } \right) }{\mathcal {B}\left( \frac{1}{2}, \alpha \right) }\times {}_2F_1\left( \frac{n-k-\gamma -1}{2}, \frac{n-k-\delta -1}{2}; \tilde{\alpha } + \frac{1}{2}; r_{xy.z}^2\right) , \end{aligned} \end{aligned}$$

where $\tilde{\alpha } = \alpha + \frac{n-k-\gamma -\delta -1}{2}$. For $\gamma =\delta =0$, the marginal posterior distribution of $\rho _{xy.z}$ is

22 $$\begin{aligned} \begin{aligned} \pi (\rho _{xy.z} \mid n, k, r_{xy.z}) =&\frac{(1-\rho _{xy.z}^2)^{(2\alpha + n - k - \gamma - \delta - 3)/2}}{\mathcal {B}\left( \frac{1}{2}, \alpha + \frac{n-k-\gamma -\delta -1}{2}\right) \phantom {.}_2F_1\left( \frac{n-k-\gamma -1}{2}, \frac{n-k-\delta -1}{2}; \alpha + \frac{n-k-\gamma -\delta }{2}; r_{xy.z}^2\right) } \\&\times \left[ \phantom {.}_2F_1\left( \frac{n-k-\gamma -1}{2}, \frac{n-k-\delta -1}{2}; \frac{1}{2}; r_{xy.z}^2\rho _{xy.z}^2\right) \right. \\&\left. + 2r_{xy.z}\rho _{xy.z} W_{\gamma ,\delta }(n-k)\phantom {.}_2F_1\left( \frac{n-k-\gamma }{2}, \frac{n-k-\delta }{2}; \frac{3}{2}; r_{xy.z}^2\rho _{xy.z}^2\right) \right] , \end{aligned} \end{aligned}$$

where $W_{\gamma , \delta } (\tilde{n})$ is defined in Ly et al. (2018, p. 7) as

23 $$\begin{aligned} \begin{aligned} W_{\gamma , \delta } (\tilde{n}) = \frac{\Gamma \left( \frac{\tilde{n}-\gamma }{2} \right) \Gamma \left( \frac{\tilde{n}-\delta }{2} \right) }{\Gamma \left( \frac{\tilde{n}-\gamma -1}{2} \right) \Gamma \left( \frac{\tilde{n}-\delta -1}{2} \right) }. \end{aligned} \end{aligned}$$

## Rewriting the trace

Using the inversion of a block matrix, the matrix $\Sigma ^{-1}$ can be rewritten in a block form as:

$$\begin{aligned} \Sigma ^{-1} = \begin{bmatrix} \Sigma _{11.2}^{-1} & -\Sigma _{11.2}^{-1}\Sigma _{12}\Sigma _{22}^{-1} \\ & \\ -\Sigma _{22}^{-1}\Sigma _{21}\Sigma _{11.2}^{-1} & \Sigma _{22}^{-1} + \Sigma _{22}^{-1}\Sigma _{21}\Sigma _{11.2}^{-1}\Sigma _{12}\Sigma _{22}^{-1} \end{bmatrix}. \end{aligned}$$

Similarly, we can write *S* as a block matrix:

$$\begin{aligned} S = \begin{bmatrix} S_{11.2} + S_{12}S_{22}^{-1}S_{21} & S_{12} \\ S_{21} & S_{22} \end{bmatrix}. \end{aligned}$$

Assuming $|S_{22}| > 0$, we can expand the trace inside of the kernel of the multivariate normal distribution, and rearrange the terms using the cyclic property of traces:

24 $$\begin{aligned} \text {tr}(S\Sigma ^{-1}) =&+ \text {tr}\left( S_{11.2}\Sigma _{11.2}^{-1}\right) \nonumber \\&+ \text {tr}\left( S_{22}\Sigma _{22}^{-1}\right) \nonumber \\&+ \text {tr}\left( S_{12}S_{22}^{-1}S_{21}\Sigma _{11.2}^{-1}\right) \nonumber \\&+ \text {tr}\left( -S_{21}\Sigma _{11.2}^{-1}\Sigma _{12}\Sigma _{22}^{-1}\right) \nonumber \\&+ \text {tr}\left( -S_{12}\Sigma _{22}^{-1}\Sigma _{21}\Sigma _{11.2}^{-1}\right) \nonumber \\&+ \text {tr}\left( S_{22}\Sigma _{22}^{-1}\Sigma _{21}\Sigma _{11.2}^{-1}\Sigma _{12}\Sigma _{22}^{-1}\right) \nonumber \\ =&+ \text {tr}\left( S_{11.2}\Sigma _{11.2}^{-1}\right) \nonumber \\&+ \text {tr}\left( S_{22}\Sigma _{22}^{-1}\right) \nonumber \\&+ \text {tr}\left( S_{22}^{-1}S_{21}\Sigma _{11.2}^{-1}S_{12}\right) \nonumber \\&+ \text {tr}\left( -S_{22}^{-1}S_{21}\Sigma _{11.2}^{-1}\Sigma _{12}\Sigma _{22}^{-1}S_{22}\right) +\nonumber \\&+ \text {tr}\left( -\Sigma _{22}^{-1}\Sigma _{21}\Sigma _{11.2}^{-1}S_{12}\right) \nonumber \\&+ \text {tr}\left( \Sigma _{22}^{-1}\Sigma _{21}\Sigma _{11.2}^{-1}\Sigma _{12}\Sigma _{22}^{-1}S_{22}\right) , \end{aligned}$$

There are now six traces in the expression. The first two traces involve the conditional variance-covariance matrix $\Sigma _{11.2}$ and the variance-covariance matrix $\Sigma _{22}$ of the controlling variables, and their sample counterparts. The latter four traces involve the cross-correlations between the controlled and controlling variables. After rearranging the terms in Eq. (24), the matrices inside of the four traces have the same dimensions, and so can be collected in a single trace, using the linearity property of traces. We can rearrange the terms further and factor out common terms to obtain the final quadratic expression:

$$\begin{aligned} \begin{aligned}&= \text {tr}(S\Sigma ^{-1}) - \text {tr}\left( S_{11.2}\Sigma _{11.2}^{-1}\right) - \text {tr}\left( S_{22}\Sigma _{22}^{-1}\right) \\&= \text {tr}\left( S_{22}^{-1}S_{21}\Sigma _{11.2}^{-1}S_{12} -S_{22}^{-1}S_{21}\Sigma _{11.2}^{-1}\Sigma _{12}\Sigma _{22}^{-1}S_{22}\right. \\&\quad \left. -\Sigma _{22}^{-1}\Sigma _{21}\Sigma _{11.2}^{-1}S_{12} + \Sigma _{22}^{-1}\Sigma _{21}\Sigma _{11.2}^{-1}\Sigma _{12}\Sigma _{22}^{-1}S_{22}\right) \\&= \text {tr}\left( (S_{22}^{-1}S_{21}\Sigma _{11.2}^{-1} - \Sigma _{22}^{-1}\Sigma _{21}\Sigma _{11.2}^{-1})(S_{12} - \Sigma _{12}\Sigma _{22}^{-1}S_{22})\right) \\&= \text {tr}\big ((S_{22}^{-1}S_{21} - \Sigma _{22}^{-1}\Sigma _{21})\Sigma _{11.2}^{-1}(S_{12}S_{22}^{-1} - \Sigma _{12}\Sigma _{22}^{-1})S_{22} \big ) \\&= \text {tr}\big (S_{22}(S_{12}S_{22}^{-1} - \Sigma _{12}\Sigma _{22}^{-1})'\Sigma _{11.2}^{-1}(S_{12}S_{22}^{-1} - \Sigma _{12}\Sigma _{22}^{-1}) \big ). \end{aligned} \end{aligned}$$

This quadratic expression replaces the four terms involving the cross-correlations:

$$\begin{aligned} \text {tr}(S\Sigma ^{-1})&= \text {tr}(S_{11.2}\Sigma _{11.2}^{-1}) + \text {tr}(S_{22}\Sigma _{22}^{-1}) + \text {tr}\big [S_{22}(S_{12}S_{22}^{-1} \\&\quad - \Sigma _{12}\Sigma _{22}^{-1})'\Sigma _{11.2}^{-1}(S_{12}S_{22}^{-1} - \Sigma _{12}\Sigma _{22}^{-1}) \big ]. \end{aligned}$$
